# Emerging Psychosis and the Family

**DOI:** 10.5402/2012/219642

**Published:** 2012-04-23

**Authors:** Martin Hambrecht

**Affiliations:** Klinik für Psychiatrie, Psychosomatik und Psychotherapie, Agaplesion Elisabethenstift Evang. Krankenhaus gGmbH, Landgraf-Georg-Straße 100, 64287 Darmstadt, Germany

## Abstract

Schizophrenias hold a special position among psychotic disorders. Schizophrenias often start in early adulthood and bear considerable psychosocial risks and consequences. Several years of nonpsychotic clinical signs and symptoms and growing distress for patient and significant others may pass by before definite diagnosis. Young males in particular often experience their first episode while still living in their primary families. Thus, the whole family system is involved. In worldwide initiatives on early detection and early intervention, near-psychotic prodromal symptoms as well as deficits of thought and perception, observable by the affected person himself, were found to be particularly predictive of psychosis. Various psychological and social barriers as well as ones inherent to the disease impede access to affected persons. Building trust and therapeutic alliance are extremely important for counseling, diagnostics, and therapy. The indication for strategies of intervention differs from the early to the late prodromal stage, depending on proximity to psychosis. For psychotherapy versus pharmacotherapy, the first evidence of effectiveness has been provided. A false-positive referral to treatment and other ethical concerns must be weighed against the risks of delayed treatment.

## 1. Introduction

The term “psychose” was coined in 1841 (C. F. Canstatt), and its meaning has changed several times since then. With the introduction of the present classification systems of ICD-10 and DSM-IV, the definition became more precise. Today, the term “psychosis” means disorders which center around delusions, hallucinations, or “severely disorganized behaviors.” Delusions are defined as impossible beliefs, which the affected person holds on to without an option for correction. Hallucinations refer to “perceptions without an object” in any sensory modality. The category that is most difficult to define concerns the “disorganized behaviors,” that make no sense (like in hebephrenia), are erratic, or inadequate for a particular situation.

Psychotic symptoms occur in schizophrenia as well as in affective, substance-related, and organic mental disorders. Diseases of the central nervous system, however, are a rare cause for psychotic symptoms in young people (e.g., in single cases of multiple sclerosis). Brain imaging and other additional diagnostics are nevertheless necessary in first psychotic episodes but mostly without pathological results. The most frequent disorders with psychotic symptoms in this age group belong to the group of schizophrenias or are induced by psychotropic substances such as cannabis and hallucinogens, or they are psychotic depressions or manias, thus belonging to the affective psychoses. 

In adolescents and young adults, diagnoses are particularly uncertain and may vary considerably across several episodes of the disease. For instance, changes in diagnosis may occur in cases where symptoms of mania, severe depression, and schizophrenic thought disorders alternately emerge in the back- and foreground of the clinical picture, and drugs may also come into play. While the prognosis in affective disorders and in substance-induced psychosis (followed by abstinence) is usually good, schizophrenic disorders always carry the risk of a chronic course and permanent social disability, placing a particularly heavy burden on the person and the family [1]. Therefore, the paper will now focus on the schizophrenias—albeit keeping the diagnostic uncertainty in mind.

**Case Vignette “Our Son Has Altered So Much”**

Reluctantly, the 21-year-old son is brought in by his parents. They are deeply concerned. For one year, he has hardly ever left the house. During day he sleeps, at night he sits at his computer. Sometimes voices can be heard from his room. Is he talking with himself? At work there has been some quarrelling, and the young man has been absent with increasing regularity. Finally, he quit the job. He did not care anymore for his sports team or his friends. His physical appearance, clothing, personal hygiene, and so forth, have changed too.

## 2. Epidemiology

The lifetime incidence of schizophrenic psychoses is about 1%, with an onset in males peaking in late adolescence and early adulthood. In females, the age of onset extends far into the third decade of life (more flat, extended distribution). On average, first onset in females is about five years later than in males. This often results in a higher rate of completed education and advanced vocational career, independent living, and more stable social integration in females with schizophrenia. Male patients, in contrast, are younger, more often live with their parents, have not completed school or vocational training, and lack own income. Therefore, the likelihood of clashes and conflicts is much higher in families with males suffering from schizophrenia. On the other hand, female patients more often have to face marital friction, separation, or divorce.

Schizophrenia brings about high illness costs in a number of respects. A considerable portion of patients suffer from lifelong disability beginning early on in life. At least one-third cannot care for themselves financially. About every tenth patient commits suicide. Two-thirds of family caregivers suffer psychologically themselves. For the economy, direct cost of treatment and care for people suffering from schizophrenia or its resulting disability is estimated to be about 4 billion euros per year (Germany).

## 3. Emerging Psychosis

Not only because of these consequences did interest in early detection and early intervention in schizophrenic disorders grow tremendously since the early 1990s [2, 3]. Long-term studies challenged the pessimistic balance previously fostered by Kraepelin's notion of schizophrenia as “dementia praecox.” With the help of new sophisticated methodology, studies focusing on the initial manifestation of the disease were able to define the onset of schizophrenia more precisely. During the last decades the introduction of effective treatment options as well as new methods of relapse prevention were of major importance, too, for early detection coming into focus.

Social withdrawal, functional deficits “Knick in der Lebenslinie*”*, suspiciousness, and depressed mood are more or less subtle but important hints for a first episode of schizophrenia. In the beginning, psychopathology is often detected only incompletely. Many affected persons do not understand what is happening to them. They are unable to describe what they experience. Various stage models, for example [4], have contributed to a general description of the early phases of schizophrenia. All models converge to the statement that the disorder develops in stages beginning with an accumulation of unspecific, “pseudoneurotic,*”* or negative symptoms. Once the climax, marked by a delusional mood, is reached, transition to an acute psychosis may occur.

In a large representative sample of persons with first episodes of schizophrenia, psychotic symptoms had been present for over one year, and in 75% of the cases nonpsychotic prodromal symptoms had been present for more than five years before the correct diagnosis had been established [5, 6]. Patients reported retrospectively that everything had started with nervousness, depressed mood, anxiety, worrying, lack of energy, and impaired daily functioning. In these prodromal states, the personal and social development is already severely delayed [7]. Various studies demonstrated that a longer duration of untreated psychosis or untreated illness goes along with delayed remission and longer hospitalization. Longer prodromal states were related to worse long-term courses, more negative emotions within the family, substance misuse, delinquency, depression, and suicidality. These correlations, however, do not prove a causal link between the duration of untreated psychosis and unfavourable later course.

## 4. Early Detection

A diagnosis of schizophrenia can best be secured when “first-rank symptoms*”* according to Schneider [8] are already present (reviewed by [9]).

- Auditory hallucinations: heard commenting or holding dialogues (or giving commands, according to Gerd Huber).
- Delusional perceptions: correct observations are interpreted within a delusional framework.
- Experiences of physical influences and anything else caused or influenced by others in the realm of feeling, striving, and will.
- Withdrawal, broadcasting, influencing, or dispersion of own thoughts.

Sometimes clear-cut first-rank symptoms are hard to confirm. They do not aid in early detection, since their appearance would already indicate the full manifestation of the disorder. Attenuated or very brief prodromes of these symptoms, however, are perceptible by the patient himself and may be used for early recognition. Frequent, although not sufficiently specific, symptoms of these prodromes are as follows.

- social withdrawal and self-isolation,
- lack of achievement, with no identifiable cause, in tasks formerly fulfilled with ease
- peculiar imaginations or strange thoughts that influence behavior (e.g., superstition, telepathy, magic, clairvoyance).

These symptoms are often accompanied by rumination and a previously unknown philosophizing about oneself, God, and the world. Delusions of persecution typically emerge as hallucinations are “explained,” for example, coenesthetic hallucinations with bizarre sensations in the own body.

The emergence of early psychosis was the major subject of the prospective Cologne Early Recognition Study [10] which analyzed the prognostic value of the so-called “basic symptoms” according to Gerd Huber. High prospective relevance was found for self-perceived abnormalities of thought, of speech perception, of discrimination between ideation and reality, derealization, nonpsychotic ideas of reference, and disturbances of perception. A person, who shows these symptoms to a defined extent, carries a more than 90% risk of developing schizophrenia within the following 10 years.

Up until now, distinct biological markers for schizophrenia are not available, but in many cases subtle psychopathology combined with careful family history leads to a reasonable prediction. Symptoms are more difficult to understand in cases with a longer, that is, child history of abnormal behaviors, for example, attention deficit syndromes, autism, conduct disorder, or social phobia in childhood. These abnormalities are usually part of neurotic or personality disorders, for instance, of obsessive-compulsive disorder or schizotypal, schizoid, paranoid, or other personality disorder.

## 5. Difficulties in Approaching Affected Persons

A number of problems interfere with the access to diagnostic assessment and treatment of persons in early psychoses. The obstacles are due, in part, to the disease, to influences of the social setting, the family, and biography, or to unfavourable previous experiences.

The capacity to relate socially to others is often impaired in these disorders. Suspiciousness and social withdrawal are among the earliest symptoms. Eugen Bleuler described “autism” as one of the core symptoms of the disease. In present terminology, the disturbances with regard to relating socially are assigned to the negative symptoms, which precede acute psychotic (“positive”) symptoms in many cases.

Adolescence is a phase of life with particularly high uncertainty. Adolescents search for orientation in this terminal stage of personality development. Finding one's place within the social setting is a core issue. Here, it becomes important to establish a stable image of oneself, the self-concept. Where do I stand, who am I, what is my position in family, at school, at work, among my peers, in society in general? The most important orientation is not provided by the family any more but by the peers. The affected person is highly dependent on acceptance within their peer-group, in which he does not want to attract unpleasant attention. Sensing differences between oneself and others leads to fear of exclusion. To be a “psycho” or worse a “schizo” is regarded as particularly mortifying. Therefore, people in early stages of psychotic disorders desperately struggle to achieve normality.

Stigmatization and self-stigmatization are closely related to this situation in adolescence. The perception of and then the labelling of a difference always form the first step to stigma (“this person is different” and “I am different”). Being different provokes negative prejudice and stereotypes, which finally result in exclusion and discrimination. At onset, mentally ill individuals look at themselves with the same derogatory prejudice and stereotypes that are popular in society. Because of this self-stigmatization, they attempt as long as possible to ignore, fend off, or deny being different. Even before the onset of the illness, affected people have often internalized the same negative attitudes that are prevalent in their community. At onset, they adopt the same attitudes against themselves. Self-stigmatization and anticipated stigmatization by others reduce patient insight into treatment and bring about more terminations of therapy.

In addition, psychotic disorders typically come along with a lack of insight which is considered to be a constitutional characteristic of delusional beliefs. This again emphasizes the importance of early detection—before the emergence of fixed delusions.

Adolescents at risk often face school failure, instability of affect, substance misuse, delinquency, and other antisocial behaviors. This promotes negative experiences with authorities in family, school, and the workplace, resulting in depreciative attitudes that are generalized on to therapists, counsellors, physicians, and so forth, who “have to” be seen. Therefore, these adolescents refuse the professional medical system and search for access to isolated subcultures, for example, in the marijuana milieu. Belonging to these peer-groups can be a relief—at least in the beginning.

There is a particularly great psychological strain on family members in cases where cannabis is persistently misused in order to overcome feelings of personal emptiness and tension. The familial context of young persons in prodromal stages is often both burdened and a burden. Parents and close relatives are perplexed, helpless, and understress. Familial tension increases. At first, this can result in overactivity, which may lead to increased pressure on the affected person, random help-seeking behavior, a myriad of partly contradicting advice, and so forth. If these activities are not successful, resignation among family members follows.

In every tenth patient with schizophrenia there is a positive family history for psychotic disorders and to a slightly higher degree for other psychiatric disorders as well. Therefore, a number of prodromal cases remember themselves or have heard stories of persons with an established disorder who might have experienced prejudice, exclusion, stigma, and bad living conditions. More or less conscious associations foster anxiety, defense, and avoidance behavior.

An accumulation of “difficult” personalities in families with prodromal and/or diagnosed cases is related to this issue of positive family history. Some family members carry personality traits such as those typical for cluster A (DSM-IV) personality disorders: hostility as the basic social attitude as found in paranoid personality disorder, deficits in social contact common to schizoid personality disorders, or peculiar habits typical for schizotypal personality disorders. Under familial stress resulting from early psychosis, these preexisting behavioral dispositions are activated and now influence not only the interaction of the family members with the affected person and the disorder but also the acceptance of treatment options. In comparison, relatives' reactions to a depressed family member (also occurring more frequently in families with psychotic patients) are usually not as complicated, albeit no more supportive.

This set of unfavourable factors has severe consequences on primary care and counselling, first assessment, and entering treatment. Many affected persons only seek help when a high degree of suffering has been reached, for example, severe anxiety or sleep disturbances. They avoid the barrier to mental health services and prefer to consult their family doctor, medical practitioners, and other paramedical professionals. Many are pushed by their cosuffering relatives. They have no motivation themselves and withdraw after the first contact or search for excuses for not showing up again. In consultations, complaints are often talked away, rationalized, or trivialized.

## 6. Recommendations for the Access to Affected Persons

Intensive public awareness campaigns generated promising results for early detection initiatives in the community but yielded open questions, too, as reviewed by [11]. On the societal as well as the individual and familial level, many obstacles to identify and to engage persons at risk for psychosis must be overcome.

Building a good and trusting relationship is the first and core objective in assessment and treatment—prior to and above subtle diagnostic procedures or even therapeutic attempts. Supporting cooperation holds the highest priority in all communications with a help-seeking person. A schematic approach is not recommended but rather a very individualized and pragmatic approach.

This means attending to those symptoms and problems that are most distressing to the affected person. This frequently concerns sleep disturbances, anxiety, depression, avoidance behavior, disturbance in social relations, cognitive deficits, misuse of cannabis or alcohol, and conflicts in family, school, and at work [12]. It is important, therefore, to determine early on the reasons why someone is seeking help or has been brought in for a consultation. Reviewing this motivation together every once in a while has been found to be useful in building adherence.

Prejudices are frequent as well as preliminary or selected pieces of knowledge about mental disorders, psychiatry, psychotherapy, and so forth. This (partly mis-)information and expectations of affected persons and their social backgrounds must be incorporated in the treatment. It makes sense to demonstrate willingness to talk about these beliefs by asking open questions. The conversation should by no means be impaired or cut off by dogmatic statements. Premature statements and confrontations are similarly inappropriate. The interviewer should not try to give brilliant predictions or produce shocking effects but to present himself as an open person and (on demand) a helpful counsellor.

Potential influences of the early symptoms, of the resulting living circumstances, and of commencing counselling and treatment on the person's self-concept must be considered, particularly, in this special phase of life of late adolescence. The person is confused. Stigmatizing influences from the social field and self-stigmatizing attitudes should be taken into consideration. In some cases, these influences have to be addressed actively. In cases with high confusion, stabilization by means of empathy and acceptance in a resource-based approach is necessary.

In many affected families, stress has already been present prior to the manifestation of psychosis. Stress has been caused by early symptoms, premorbid deficits as well as unfavourable reactions, and communication patterns within the family. Conflicts create strain on the family atmosphere and might be brought into consultation and treatment. Professional caregivers should beware of being exploited by family members. Coalitions with relatives should be avoided. In the treatment of early psychosis, the most important but often most fragile relationship is the relationship with the affected person.

Because of early cognitive deficits, for example, in attention and memory that can be part of the prodrome [13], an adaptive style of communication is recommended, that is, simple and clear messages. Information should be easy to understand, without foreign words, and sufficiently redundant. The social setting around assessment, counselling, and treatment should be straightforward, relaxed, calm, and without distractions.

In other settings, a no-tolerance stance on addictive substances may be appropriate. In early psychosis, however, this attitude will cut off access to many affected persons. Cannabis misuse is universal and has been found to be twice as frequent in prodromal patients than in their peers [14]. The same holds true for alcohol misuse but to a lesser degree. Problems of substance misuse must be dealt with in a flexible way during the early phase of treatment. The basic steps should center on information, assessment, and a dialogue as elaborated in “motivational interviewing” [15], promoting empathy, enforcing self-efficacy, providing support in finding new solutions oneself, and creating discrepancies. At his point, the interview elaborates contradictions between own wishes, goals, and expectations on the one side, and the actual behavior and its consequences on the other side. For example, a cannabis-consuming adolescent boy expresses his wish to share experiences with peers, but during the interview he becomes aware that the real effect of the misuse is social withdrawal and separation.

If the barrier to see a psychologist, psychotherapist, or psychiatrist is too high, the family doctor must be involved [16]. Profound and long-lasting knowledge of the family and its members helps to detect subtle changes and build on favorable communication patterns of the past.

## 7. Evaluated Therapeutic Concepts

As in therapy and relapse prevention, the vulnerability-stress-coping model of schizophrenia is considered to be the framework of early intervention in schizophrenia. Therefore, the fundamental principles are the following: raising the vulnerability threshold, reducing stress and stress reactivity, and strengthening protective factors in persons at risk. All preventive strategies need to be adapted to the individual's level of risk for psychosis, to the stage of the disorder, to the individual's expectations, preferences, resources, and current symptoms, and to comorbid conditions like substance misuse.

In relapse prevention of schizophrenia, the effectiveness of various pharmacological, psychotherapeutic, and psychosocial methods has been demonstrated. These include antipsychotic medication, psychoeducation, stress management, cognitive behavioral therapy, and other types of psychotherapy, including family interventions. As an analogy to relapse prevention, we expect that these methods also work in early intervention. The combination and integration of several strategies were found to be beneficial, but poor treatment adherence and cooperation must be expected in early intervention just as in relapse prevention.

Current knowledge on early intervention relies on a few randomized controlled trials (RCT) and some case series as well as clinical experience, as summarized by [17].

In Melbourne's PACE Clinic [18] persons at ultra high risk (i.e., very close to psychosis) were included in an RCT. They reported either brief, limited, intermittent psychotic symptoms (BLIPS), attenuated psychotic symptoms, or the combination of a familial or perinatal risk in combination with acute deficits in psychosocial functioning. While the intervention group received a combination of cognitive behavioral therapy, social support, and up to 2 mg of risperidone, controls only received supportive visits. There were only 10% of transitions to psychosis in the intervention group (of all cases with complete therapy adherence), whereas 36% of the controls had a transition to psychosis.

In Germany, two early intervention studies were conducted within the Competence Network on Schizophrenia. Ethical as well as clinical considerations suggested to split the prodromal development of schizophrenia into two stages: within the early prodromal stage (further away from psychosis) therapy should rely on psychological methods, and within the late prodromal stage (close to psychosis) therapy is built around medication. Inclusion criteria for the early prodrome state were the above-mentioned basic symptoms, which were found to be predictive for transition in the study by Klosterkötter et al. [10], that is, self-observed abnormalities of thought, perception, and self-experience. Persons with a preexisting risk (familial or perinatal history), who had recently a drop in their psychosocial functioning, were also included in the group further away from psychosis. After randomization, the intervention group received manualized individual and group therapy, computerized cognitive training, and the offer of family counseling, all in all lasting about one year [19]. Controls were only accompanied supportively. Bechdolf et al. [20] reported that the treatment group had 5% transitions within 12 months, whereas the control group had 15% transitions to a late prodrome or to psychosis (*P* < 0.1).

Similar to the Melbourne studies, the German Competence Network used a definition of the late prodromal state that is based on brief, limited, intermittent psychotic symptoms, or attenuated psychotic symptoms. In this RCT, the controls received clinical management as usual, while the intervention group additionally received amisulpride, which was cautiously titrated up until the symptoms had disappeared (mean final dosage 188 mg amisulpride). Within 6 months, 5% transitions to psychosis occurred in the intervention group and 21% in the control group. Active treatment also improved psychosocial functioning (GAF Score), whereas attenuated psychotic symptoms, basic symptoms, and depressive symptoms decreased markedly [21].

A summary on early intervention should note that a number of findings demonstrate the effectiveness of low-dose atypical antipsychotics for prevention for patients close to psychosis [22]. Specially designed psychological therapy appears to work in early prodromes. These conclusions, however, rely on only a few studies which were carried out with strong dedication and broad resources in specialized research settings.

With this incomplete empirical foundation in mind, the ethical aspects of early detection and early intervention should be taken into consideration. Wrong diagnoses, that is, particularly false-positive predictions must be addressed as well as undesirable and side effects of assessment and treatment. These concerns are counterbalanced by the fact that patients and/or relatives seek help themselves, because they are already suffering considerably from existing symptoms. A “wait-and-see” approach is not without risk either. The consequences of untreated long-lasting early psychosis were already described.

The focus of early detection should always be on cautious information and careful balancing of the pros and cons of the treatment options. Interventions must be tailored individually to the complaints and should employ supportive psychological training methods before other kinds of interventions. The indication for medication should be examined carefully. Only low-dose novel antipsychotics with very low levels of side effects should be considered.

## 8. Core Recommendations for Clinical Practice

*Building a Trustful Working Alliance*. (i) I record and label the individual burden and address the most urgent problems. (ii) Although I acknowledge the relatives' distress, the affected person continues to be my most important ally. (iii) I do not override the help-seeking person, but I slowly approach the subjective side of the symptoms, previous experience, knowledge, and so forth.

*Assessment*. (i) I frame the careful diagnostic exploration calmly and avoid evaluative comment. (ii) Recently emerging self-perceived abnormalities of thought, perception, and self-experience are particularly helpful in the assessment (i.e., basic symptoms according to G. Huber).

*Therapeutic Intervention*. (i) My strategy is supportive, problem-solving, and resource oriented. (ii) In advanced prodromal states close to psychosis, I take low-dose antipsychotic medication into consideration.

## Appendix

### Predictive Basic Symptoms

The following symptoms are reported by patients as self-observed complaints and are assessed with the *Bonn Scale for the Assessment of Basic Symptoms* [23]. Their predictive values were reported by [10].

Interference of thoughts:
  Thinking is often disturbed by other emotionally neutral thoughts that intrude into the train of thought.
  False-positive predictions: 4%.
  False-negative predictions: 29%.
Perseveration of thoughts:
  Personal, emotionally neutral thoughts are repeating, similarly to obsessions.
  False-positive predictions: 6%.
  False-negative predictions: 34%.
Pressure of thoughts:
  Personal, emotionally neutral thoughts are flooding intentional thinking.
  False-positive predictions: 2%.
  False-negative predictions: 31%.
Blocking of thoughts:
  Lines of thought often bread off or are interrupted again and again by emotionally neutral thoughts.
  False-positive predictions: 7%.
  False-negative predictions: 33%.
Deficits of receptive speech:
  The comprehension for read or spoken information is markedly reduced.
  False-positive predictions: 4%.
  False-negative predictions: 30%.
Disturbed discrimination of imagination and perception:
  The differentiation between real experiences and objects from one's own fantasies often fails.
  False-positive predictions: 3%.
  False-negative predictions: 36%.
Derealization
  The environment appears unreal and strangely alien.
  False-positive predictions: 5%.
  False-negative predictions: 36%.
Nonpsychotic disturbances of visual or acoustic perceptions:
  Strange abnormalities of sight or hearing (similarly of smell, taste, touch, etc.) recurrently occur, for which a physical cause cannot be found.
  False-positive predictions: 8%/6%.
  False-negative predictions: 27%/35%.
Nonpsychotic ideas of relatedness (subject centrism):
  Random events are constantly related to oneself, although they have nothing to do with oneself. The person, however, is able to distance himself from this impression quickly.
  False-positive predictions: 6%.
  False-negative predictions: 30%.
